# Self-Inflicted Hyperkalemia: A Case Report

**DOI:** 10.1155/2012/458049

**Published:** 2012-05-29

**Authors:** Nathaniel Lisenbee, Bobby Desai, Michael Falgiani

**Affiliations:** Department of Emergency Medicine, College of Medicine, University of Florida 1329 SW 16th Street, P.O. Box 100186, Gainesville, FL 32610-0186, USA

## Abstract

We present a case of a potentially lethal ingestion of potassium pills. After presentation, we briefly review the epidemiology and pathology of hyperkalemia.

## 1. Case Presentation

The patient is a 38-year-old male that was transferred to our emergency department (ED) from an outside hospital status post cardiac arrest. The patient was an inmate at a local prison who was found unresponsive in his prison cell. Emergency Medical Services (EMS) was called for medical support. Minimal health information was provided by the prison. The patient was found by EMS to be in ventricular tachycardia with a palpable pulse and was intubated on the scene. The patient was given 150 mg of amiodarone, and transportation to an outside facility was initiated. On laboratory evaluation at the outside hospital, the patient was noted to have a potassium level of 9. Treatment of the patient's hyperkalemia with insulin, beta-agonists, and calcium was initiated. After initial resuscitation, the patient was transported to our ED. While en route, the patient experienced an episode of pulseless ventricular tachycardia, requiring two episodes of cardioversion. Return of spontaneous circulation was achieved prior to ED arrival.

Upon arrival to our ED, vitals were as follows: blood pressure 91/48, heart rate 145 beats per minute, respiratory rate 14 breaths per minute, oxygen (O_2_) saturation 99%, temperature 35.3 Celsius (C). An electrocardiogram (ECG) was performed, which documented ventricular tachycardia at a rate of 140 without ST segment or T wave changes. On exam, the patient was noted to be intubated. Head, ears, eyes, nose, and throat (HEENT) exam revealed 3 millimeter (mm) pupils that were sluggishly reactive to light and equal bilaterally and an endotracheal tube at 25 centimeters (cm) at the teeth. The remainder of the HEENT exam was unremarkable. Neck exam revealed a cervical collar in place with no cervical spine step-off noted. Cardiovascular exam revealed a heart rate of 140 beats per minute with palpable distal pulses. Lungs were clear to auscultation bilaterally. Abdomen was soft with good bowel sounds and no evidence of distension. On neurological exam, the patient was noted to be sedated with a Glasgow coma scale (GCS) of 3T.

The patient had blood sent to the lab for multiple studies. The patient's complete blood count (CBC) revealed a white blood cell count of 23.6 thou/cu mm, a hemoglobin of 14.7 g/dL, a hematocrit of 45.5%, and a platelet count of 181 thou/cu mm. His basic metabolic panel revealed a sodium of 143 mmol/L, a potassium of 8.7 mmol/L, a chloride of 120 mmol/L, a bicarbonate of 16 mmol/L, a BUN of 22 mg/dL, a creatinine of 1.06 mg/dL, and a glucose of 191 mg/dL. Venous blood gas revealed a pH of 7.06, pCO_2_ of 58 mmHg, and pO_2_ of 77 mmHg. International normalized ratio (INR) was 1.4. Cardiac markers and urinalysis were both unremarkable. A urine drug screen was positive for benzodiazepines.

The patient's emergency department management began with initial stabilization. Due to his persistent ventricular tachycardia, the patient was given another bolus of amiodarone upon arrival and a subsequent amiodarone infusion was started. Due to low mean arterial pressures, two liter of normal saline was given intravenously, a right subclavian central venous line was placed, and a left radial arterial line was placed. A levophed drip was started at 0.1 mcg/kg/min and titrated to maintain a mean arterial pressure (MAP) of 65 for pressure support. Treatment for hyperkalemia was readministered concurrently, consisting of insulin, beta-agonists and kayexalate. Chest X-ray revealed clear lungs with radio paque capsules in the fundus of the stomach. Over the next thirty minutes, the patient's rhythm converted to normal sinus rhythm and slowed to around 70 beats per minute. Repeat potassium lab draw revealed persistently elevated potassium level of 8.3 mmol/L, and hyperkalemia treatment was repeated. At this point, nephrology was consulted for emergent dialysis, and the medical intensive care unit (MICU) was notified for admission. We later found out, from the MICU team, that the patient had an esophagogastroduodenoscopy (EGD) performed to remove the unknown pills from the patient's stomach, which were found to be potassium chloride pills. After removal of the pills and multiple episodes of dialysis, the patient stabilized and was weaned from pressure and ventilator support. The patient's leukocytosis was believed to be a stress response as the MICU team was never able to determine a source of infection; however, the patient remained on-broad spectrum intravenous antibiotics over the course of his stay.

## 2. Discussion

Hyperkalemic cardiac arrest is a severe but not uncommon complication of hyperkalemia. Under normal physiologic conditions, only 1% to 2% of the body's total potassium is extracellular. As potassium plays a critical role in cellular depolarization and tissue excitability, minor fluctuations in extracellular levels can have significant pathologic effects, potentially leading to cardiac dysrhythmias and cardiac arrest. Hyperkalemia is most commonly caused by renal failure or medication side effects; however, many cases are multifactorial [1]. It is important to be able to recognize hyperkalemia and to be familiar with its treatment as hyperkalemia affects as many as 8% of hospitalized patients [2].

While the correlation of characteristic electrocardiogram findings with hyperkalemia is poor, it is still important to be able to recognize the characteristic electrocardiogram progress as hyperkalemia progresses [1]. The pattern of ECG findings with hyperkalemia depends on serum K^+^ levels, with initial ECG changes being noted at serum concentrations between 5.5 and 7.0 millimoles/liter (mmol/L) and serious, potentially fatal arrhythmias not being seen until serum potassium concentrations approach 10.0 mmol/L [2]. Initially in hyperkalemia, the QT interval shortens and T waves take on a characteristic peaked appearance. As the hyperkalemia progresses, the PR interval and the QRS length increase. At this point, P waves may disappear and conduction disturbances may be noted. Ultimately, the QRS will continue to widen due to increasing conduction delay and exhibit a “sine wave” pattern, which is a sign of impending cardiac arrest [2].

Hyperkalemia management consists of multiple treatments. Insulin (given with glucose) and beta-agonists act quickly to relocate potassium intracellularly. Exchange resins, such as kayexalate, bind potassium in the gut and lead to its removal from the body in the stool. Loop and thiazide diuretics increase potassium excretion in the urine. Calcium provides benefit in that it increases membrane stability and antagonizes the destabilizing effects of hyperkalemia on cellular membranes. Bicarbonate use is controversial; however the theoretical effect is due to the increase in serum pH, which leads to activation of the H^+^/K^+^ exchange pump, causing shift of potassium intracellularly [3]. In regards to hyperkalemia from oral potassium ingestion, as seen in this case presentation, another potential option for treatment is whole bowel irrigation, which can be very helpful when a patient is seen to have a large pill burden in their stomach [4]. Ultimately, cases of hyperkalemia can be improved with hemodialysis, especially those involving hemodynamic instability or those resistant to standard therapies [5].

## 3. Conclusion

Hyperkalemia is an important cause of tachyarrhythmias and cardiac arrest. The differential diagnosis for hyperkalemia is varied and extensive. Clinicians should consider hyperkalemia and its differential as a possible etiology in all cardiac arrest situations. As seen in this case, clinicians should initiate therapy quickly and be mindful of potassium ingestions as a cause in cases refractory to standard therapy. Ultimately, hemodialysis is an available option in the critically ill patient.

## Abbreviations

KCl: Potassium chloride
ED: Emergency department
EMS: Emergency medical services
O_2_: Oxygen
C: Celsius
ECG: Electrocardiogram
HEENT: Head, eyes, ears, nose & throat
mm: Millimeter
cm: Centimeter
GCS: Glasgow coma scale
CBC: Complete blood count
MICU: Medical intensive care unit
EGD: Esophagogastroduodenoscopy
L: Liter
mmol: Millimole
mmol/L: Millimole/liter.
